# TyG index predicts adverse cardiovascular outcomes in patients with multimorbidity of hypertension and obstructive coronary artery disease: a cohort study

**DOI:** 10.3389/fcvm.2026.1861084

**Published:** 2026-07-16

**Authors:** Qingying Jiao, Mengmeng Wang, Zishan Liu, Zhiyi Yu, Guixia Sun, Jiachao Xu, Tianqi Teng, Yanyan Du, Zihan Dong, Yongqi Shan, Jingjing Zhang, Zihan Sun, Haichu Yu

**Affiliations:** 1 Department of Cardiology, The Affiliated Hospital of Qingdao University, Qingdao, Shandong, China; 2 Qingdao Traditional Chinese Medicine Hospital, Qingdao Hiser Hospital Affiliated of Qingdao University, Qingdao, Shandong, China; 3 The Affiliated Hospital of Qingdao University, Qingdao, Shandong, China; 4 Department of Cardiology, The Affiliated Hospital of Jining Medical University, Jining, Shandong, China

**Keywords:** hypertension, insulin resistance, major adverse cardiovascular events, Mendelian randomization, obstructive coronary artery disease, triglyceride-glucose index

## Abstract

**Background:**

Whether elevated triglyceride-glucose (TyG) index is linked to long-term prognosis in individuals with hypertension and obstructive coronary artery disease (OCAD) remains unclear. We applied a combination of a cohort study and Mendelian randomization (MR) analysis to investigate this relationship.

**Methods:**

This study selected 1,024 individuals with essential hypertension and OCAD who were treated at The Affiliated Hospital of Qingdao University (June 2023–April 2024). We evaluated the correlation between the TyG index and the 2-year major adverse cardiovascular events (MACE) risk using logistic regression, restricted cubic splines (RCS), subgroup analysis and interaction testing. Causal inference was conducted using MR. MACE was defined as a composite of: (1) all-cause death; (2) non-fatal myocardial infarction (MI); (3) unplanned revascularization; and (4) rehospitalization for unstable angina.

**Results:**

After a 2-year follow-up, 202 patients (19.73%) experienced MACE. After multivariable adjustment, each one-unit increment in the TyG index was independently linked with a 45.2% increased risk of MACE (odds ratio: 1.452, 95% confidence intervals 1.101–1.913; *P* = 0.008). Subgroup analysis indicated that the TyG index demonstrates consistent predictive performance across different subgroups. The TyG index exhibited a significant positive linear association with MACE, as confirmed by RCS analysis. MR proved a significant causal relationship between an elevated TyG index and the incidence of various adverse cardiovascular outcomes.

**Conclusions:**

Elevated TyG index levels were strongly linked to worse clinical outcomes in individuals with hypertension and OCAD. MR suggested a potential causal relationship between them.

## 1 Introduction

Coronary artery disease (CAD) is the predominant cause of cardiovascular death (1). Obstructive CAD (OCAD) is a core clinical subtype of CAD and the main pathological type responsible for sudden cardiac death, acute coronary syndrome (ACS), and angina pectoris (2, 3). Compared with non-obstructive CAD, OCAD carries a significantly higher risk of adverse prognosis, which is primarily attributed to the initiation and progression of atherosclerosis (4). Furthermore, hypertension, a key risk factor for atherosclerosis, frequently co-occurs with OCAD, thus posing a significant health threat (5). Emerging evidence suggests that high systolic blood pressure (SBP) ranks as the primary cause among various risk factors for ischemic heart disease (IHD) (6). A significant linear dose-response relationship was observed between SBP level and IHD risk (7). Compared with an SBP of 100 mmHg, elevations to 130, 140, and 165 mmHg were associated with 76.8%, 129.3%, and 305.5% increased risks of IHD, respectively (7). Globally, the proportion of IHD disability-adjusted life years (DALYs) resulting from high SBP was as high as 49.9% (8). This suggests that, theoretically, blood pressure control could prevent nearly half of the CAD burden. Hypertension is responsible for 1 in 3 heart attacks and stroke deaths globally (9). Among the 245 million adults with hypertension in China, the proportion of hospitalized patients with concomitant CAD was as high as 30.5% (10). The coexistence of hypertension and CAD is not merely additive but rather exerts a synergistic effect on worsening prognosis (11). Therefore, effective risk management and early screening in patients with this comorbidity constitute an urgent clinical priority.

The triglyceride-glucose (TyG) index, first introduced in 2008, has gained recognition as a reliable proxy marker of insulin resistance (IR) (12, 13). In 2016, the TyG index was first confirmed as a prognostic marker for cardiovascular disease (CVD) (14). Subsequently, studies on the relationship between the TyG index and CVD have been conducted (15). Previous studies have confirmed that the TyG index is closely associated with the development, progression, and prognosis of various CVDs, including stroke, hypertension, CAD, and heart failure (16–18). Additionally, studies have shown that the TyG index may help identify hypertensive individuals at elevated risk for myocardial ischemia (19). In their study of patients with ischemia and no obstructive coronary artery disease (INOCA), Zhang et al. found that the TyG index demonstrated a significant association with myocardial ischemia and adverse prognosis (20). In individuals with essential hypertension and OCAD, the risk of cardiovascular events is significantly elevated, underscoring the critical need for robust prognostic biomarkers to enable risk stratification. However, previous studies on the TyG index have predominantly enrolled heterogeneous populations with CAD and featured relatively short follow-up durations. Consequently, causal evidence specifically targeting the pure OCAD subgroup and long-term (2-year) outcomes remains lacking.

Mendelian randomization (MR) operates on a principle analogous to that of randomized controlled trials (RCTs), utilizing genetic variants to serve as instrumental variables (IVs) to explore potential causal relationships between exposure risk factors and health outcomes (21). This approach effectively mitigates the impact of confounding and reverse causality that may be present in observational studies (22). Several MR studies have investigated the causal relationship between the TyG index and cardiovascular diseases—for instance, hypertension, aortic dissection, and heart failure (12, 23, 24). However, evidence from MR analyses regarding the association between the TyG index and various adverse cardiovascular outcomes remains insufficient. Therefore, this study sought to investigate the relationship between the TyG index and 2-year major adverse cardiovascular events (MACE) in participants with hypertension and OCAD by integrating a retrospective cohort study with MR analysis. Our objective is to develop a more accurate risk stratification approach for this high-risk group, enabling timely identification and early intervention to prevent disease progression and reduce the burden of serious cardiovascular complications.

## 2 Methods

### 2.1 Study design and population

**Figure 1:**
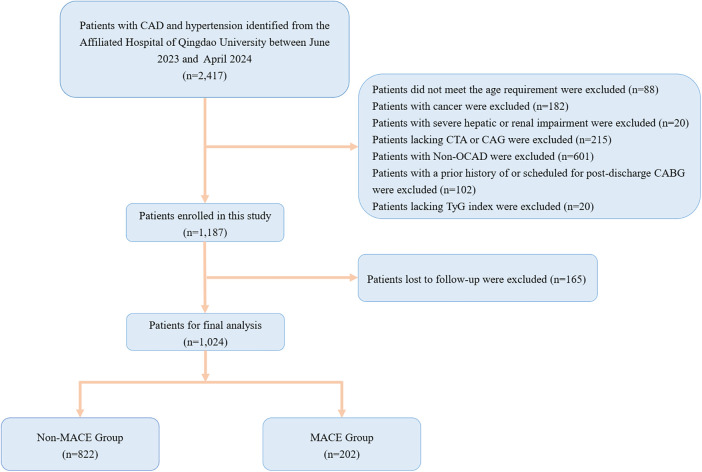
The flowchart of this study.

This study conducted a retrospective cohort study enrolling patients who were treated at The Affiliated Hospital of Qingdao University (June 2023–April 2024). A detailed flow diagram of the study is presented in Figure 1. The inclusion criteria were: (1) patients diagnosed with essential hypertension and OCAD; and (2) age between 18 and 80 years. Exclusion criteria comprised: (1) age outside the specified range; (2) diagnosis of a malignant tumor; (3) presence of severe hepatic or renal failure; (4) lack of coronary angiography (CAG) or coronary computed tomography angiography (CCTA) data; (5) diagnosis of non-OCAD; (6) prior or planned coronary artery bypass grafting (CABG) after discharge; (7) missing TyG index data; and (8) loss to follow-up. Of the 2,417 participants, 1,393 were excluded, and the final study population comprised 1,024 patients. Based on the development of MACE during the 2-year follow-up, participants were assigned to either the MACE group (*n* = 202) or the non-MACE group (*n* = 822).

This study was performed in strict compliance with the STROBE guidelines and the Declaration of Helsinki, and received approval from the Ethics Committee of The Affiliated Hospital of Qingdao University (approval number: QYFYWZLL42088). Informed consent was not required given the retrospective observational design.

### 2.2 Data selection and definition

Baseline information was collected for each patient upon admission, comprising demographic characteristics, medical history, imaging examinations and laboratory tests. Additionally, the length of hospital stay (days) was also collected. Demographic characteristics comprised: sex, age, weight, height, heart rate (HR), diastolic blood pressure (DBP), SBP, body mass index (BMI), smoking history (yes/no), alcohol consumption history (yes/no), and current residence (local or non-local); medical history documented the presence or absence of diabetes mellitus (DM); laboratory tests included: white blood cell count (WBC, ×10^9^/L), neutrophil count (NEUT, ×10^9^/L), lymphocyte count (LYMPH, ×10^9^/L), monocyte count (MONO, ×10^9^/L), platelet count (PLT, ×10^9^/L), fasting blood glucose (FBG, mmol/L), total cholesterol (TC, mmol/L), triglycerides (TG, mmol/L), low-density lipoprotein cholesterol (LDL-C, mmol/L), high-density lipoprotein cholesterol (HDL-C, mmol/L), blood urea nitrogen (BUN, mmol/L), estimated glomerular filtration rate (eGFR, mL/min/1.73 m^2^), serum creatinine (Scr, μmol/L), and N-terminal pro-brain natriuretic peptide (NT-proBNP, pg/mL). Imaging examinations included echocardiography, CCTA, and CAG. Additionally, we documented the number of coronary lesions, percutaneous coronary intervention (PCI) status, and whether the patient had a diagnosis of acute myocardial infarction (AMI). TyG index was computed using the formula: ln [TG (mg/dL) × FBG (mg/dL)/2]; TyG-BMI was subsequently calculated as TyG index × BMI (25). Alcohol consumption history was defined as no (never regular drinkers) or yes (former regular drinkers, defined as drinking at least once per week for ≥6 months) (26).

OCAD was defined as the presence of ≥50% luminal diameter stenosis in at least one major epicardial coronary artery, as detected by CCTA or CAG (27). The definition of hypertension encompassed three conditions: ongoing antihypertensive therapy, a documented history of hypertension, or a new diagnosis established by systolic blood pressure ≥140 mmHg and/or diastolic blood pressure ≥90 mmHg (28).

### 2.3 Follow-up and clinical endpoints

Physicians conducted clinical follow-up through querying the hospital's electronic medical record system or via telephone contact with patients, at a 2-year interval. An independent committee of two cardiologists, blinded to baseline data, adjudicated all endpoints.

The primary endpoint of this study was defined as the occurrence of MACE during the 2-year follow-up period. MACE was defined as (29): (1) all-cause death; (2) non-fatal MI; (3) unplanned revascularization; and (4) rehospitalization for unstable angina (UA). Unplanned revascularization comprised unplanned PCI or CABG. The secondary endpoints were the individual components of the primary endpoint.

### 2.4 MR analysis

MR analysis relies on three key assumptions (21): the relevance, exclusion restriction, and independence assumptions. (1) a strong association between the IVs and the exposure is essential; (2) the IVs should not have a direct effect on the outcome; (3) the IVs should be independent of each other and not associated with any potential confounders. The inverse variance weighted (IVW) method served as the primary approach for investigating the potential causal association between the TyG index and various adverse cardiovascular outcomes. From the UK Biobank, 192 single-nucleotide polymorphisms (SNPs) that reached genome-wide significance for association with the TyG index in prior research were selected as IVs (Supplementary Table S1). IVs were selected based on a stringent threshold of *P* < 5 × 10^−8^, with linkage disequilibrium clumping (r^2^ < 0.001) applied to guarantee the independence of the included SNPs. Data on adverse cardiovascular outcomes were obtained from various specialized datasets to ensure the robustness of the findings: UA data were obtained from ebi−a−GCST90018712, finn−b−I9_UAP, ukb−d−I9_UAP, finn−b−I9_UAP_EXNONE and ebi−a−GCST90018932; Coronary heart disease data from ieu−a−6, ieu−a−7, ieu−a−8 and ieu−a−9; MI data from ebi−a−GCST011364, ebi−a−GCST011365, ebi−a−GCST90018657, ebi−a−GCST90018877, ebi−a−GCST90038610, finn−b−I9_MI, ieu−a−798, ukb−d−I9_MI, finn−b−I9_MI_EXNONE, finn−b−I9_MI_STRICT, finn−b−I9_MI_STRICT_EXNONE, finn−b−I9_POSTAMI and finn−b−I9_POSTAMI_EXNONE; cardiac arrest data from finn−b−I9_CARDARR and finn−b−I9_CARDARR_EXNONE; all-cause death data from ukb−d−ICDMAIN_ANY_ENTRY. Further information on the datasets can be accessed at https://gwas.mrcieu.ac.uk/.

### 2.5 Statistical analysis

Continuous variables were expressed as median (interquartile range), and comparisons between groups were performed using the Kruskal–Wallis rank sum test or Mann–Whitney U test. Categorical variables were expressed as frequency (percentage), and group comparisons were conducted using Fisher's exact test or the Chi-square test.

To evaluate the associations of the TyG index and TyG-BMI with the risk of MACE, univariate and multivariable logistic regression analyses were performed. The results were presented as odds ratio (OR) with 95% confidence intervals (CI). We constructed four logistic regression models: Model 1 represented the crude analysis without adjustment for any covariates; Model 2 was subsequently adjusted for age and sex; Model 3 was additionally adjusted for DM, AMI, smoking history, alcohol consumption history, length of hospital stay, current residence, number of coronary lesions, whether PCI was performed, baseline DBP and SBP, HR, and BMI; Model 4 was further adjusted for left ventricular ejection fraction (LVEF), PLT, HDL-C, LDL-C, BUN, eGFR, and NT-proBNP based on Model 3. Potential confounders were entered into the multivariable models if they showed statistical significance in univariate analyses or were considered clinically relevant. The TyG index and TyG-BMI were included as both categorical and continuous variables. Restricted cubic splines (RCS) were used to evaluate the nonlinear relationship between the TyG index and outcome, with the same adjustment factors as those used in the logistic regression models. Moreover, we constructed receiver operating characteristic (ROC) curves to evaluate the predictive value of the TyG index and TyG-BMI for outcomes, with the area under the curve (AUC) and its 95% CI calculated using a nonparametric method. To further evaluate potential modifying effects, we undertaken subgroup analyses based on sex, age, BMI, presence of DM, diagnosis of AMI, performance of PCI, number of coronary lesions (1, 2, or multivessel), smoking history, and alcohol consumption history. Interaction testing was also performed to detect heterogeneity between subgroups.

All statistical analyses were performed using IBM SPSS Statistics (version 27.0) and R software (version 4.5.1). A two-sided *P* value < 0.05 was considered statistically significant.

## 3 Results

### 3.1 Baseline characteristics according to MACE occurrence

Table 1 presents the baseline characteristics of people with hypertension and OCAD, stratified by the occurrence of MACE. The study cohort had a median age of 67 years and was predominantly male (64.8%). Among the 1,024 patients, 202 (19.73%) experienced MACE throughout the entire 2-year follow-up period. Compared with those who did not experience MACE, the MACE group exhibited several distinct characteristics: a higher proportion of participants with DM; a greater prevalence of multivessel coronary artery disease; higher WBC and MONO counts; elevated levels of FBG, BUN, TyG index, and TyG-BMI; as well as lower baseline SBP, PLT counts and HDL-C levels. However, no significant differences were found between the two groups for the remaining variables (*P* > 0.05).

**Table 1:** Baseline characteristics of patients stratified by MACE.

Characteristic	Overall	Non-MACE	MACE	*p*-value
	*N* = 1,024	*N* = 822	*N* = 202	
Age, years	67 (60, 72)	67 (60, 72)	67 (60, 72)	0.939
Male, *N* (%)	664 (64.8)	526 (64.0)	138 (68.3)	0.249
BMI, kg/m^2^	26.00 (24.20, 28.30)	26.00 (24.10, 28.30)	26.10 (24.20, 28.73)	0.173
SBP, mmHg	137 (125, 148)	138 (126, 148)	134 (122, 146)	0.013
DBP, mmHg	78 (70, 85)	78 (71, 85)	76 (70, 83)	0.055
HR, bpm	68 (61, 76)	67 (61, 75)	69 (62, 78)	0.099
Smoking, *N* (%)	323 (31.5)	257 (31.3)	66 (32.7)	0.700
Drinking, *N* (%)	243 (23.7)	199 (24.2)	44 (21.8)	0.468
Residence, *N* (%)				0.056
Local	608 (59.4)	500 (60.8)	108 (53.5)	
Non-local	416 (40.6)	322 (39.2)	94 (46.5)	
DM, *N* (%)	396 (38.7)	300 (36.5)	96 (47.5)	0.004
AMI, *N* (%)	269 (26.3)	215 (26.2)	54 (26.7)	0.867
Number of coronary lesions, *N* (%)				0.031
One-vessel disease	99 (9.7)	85 (10.3)	14 (6.9)	
Two-vessel disease	196 (19.1)	167 (20.3)	29 (14.4)	
Multi-vessel disease	729 (71.2)	570 (69.4)	159 (78.7)	
PCI, *N* (%)	497 (48.5)	399 (48.5)	98 (48.5)	0.995
Length of hospital stay, days	4 (2, 5)	4 (2, 5)	4 (2, 5)	0.500
LVEF, %	62 (60, 64)	62 (60, 64)	62 (58, 64)	0.065
WBC, 10^9^/L	6.52 (5.47, 7.74)	6.46 (5.36, 7.73)	6.74 (5.82, 7.75)	0.047
NEUT, 10^9^/L	3.88 (3.07, 4.91)	3.86 (3.03, 4.92)	4.02 (3.20, 4.90)	0.149
LYMPH, 10^9^/L	1.83 (1.48, 2.27)	1.83 (1.48, 2.25)	1.90 (1.48, 2.34)	0.225
MONO, 10^9^/L	0.49 (0.40, 0.61)	0.49 (0.40, 0.61)	0.52 (0.44, 0.61)	0.021
PLT, 10^9^/L	217 (184, 258)	219 (185, 259)	208 (180, 246)	0.036
TC, mmol/L	4.19 (3.46, 5.05)	4.23 (3.49, 5.08)	4.06 (3.41, 4.85)	0.102
TG, mmol/L	1.24 (0.91, 1.79)	1.22 (0.91, 1.78)	1.33 (0.95, 1.87)	0.053
HDL-C, mmol/L	1.34 (1.17, 1.54)	1.35 (1.18, 1.55)	1.29 (1.14, 1.51)	0.009
LDL-C, mmol/L	2.26 (1.72, 2.99)	2.29 (1.72, 3.04)	2.13 (1.72, 2.76)	0.072
FBG, mmol/L	5.56 (4.95, 6.83)	5.49 (4.95, 6.66)	5.96 (5.04, 7.33)	0.007
BUN, mmol/L	5.95 (4.98, 7.15)	5.88 (4.93, 7.00)	6.23 (5.20, 7.90)	0.003
Scr, umol/L	95 (86, 107)	95 (86, 107)	94 (86, 107)	0.796
eGFR, mL/min/1.73 m^2^	65.90 (57.76, 74.25)	65.99 (58.01, 73.98)	65.31 (56.98, 76.96)	0.615
NT-proBNP, pg/mL	108 (45, 416)	108 (44, 405)	109 (47, 566)	0.387
TyG index	8.67 (8.31, 9.10)	8.64 (8.28, 9.08)	8.76 (8.40, 9.20)	0.006
TyG-BMI	226.64 (205.33, 250.63)	225.36 (204.31, 248.72)	230.95 (208.34, 259.96)	0.013

BMI, body mass index; SBP, systolic blood pressure; DBP, diastolic blood pressure; HR, heart rate; DM, diabetes mellitus; AMI, acute myocardial infarction; PCI, percutaneous coronary intervention; LVEF, left ventricular ejection fraction; WBC, white blood cell; NEUT, neutrophil; LYMPH, lymphocyte; MONO, monocyte; PLT, platelet; TC, total cholesterol; TG, triglycerides; HDL-C, high-density lipoprotein cholesterol; LDL-C, low-density lipoprotein cholesterol; FBG, fasting blood glucose; BUN, blood urea nitrogen; Scr, Serum Creatinine; eGFR, estimated glomerular filtration rate; NT-proBNP, N-terminal pro-B-type natriuretic peptide; TyG index, Triglyceride-glucose index.

### 3.2 Baseline characteristics by TyG index quartiles

Table 2, Supplementary Table S2, Figure S1 summarized the baseline characteristics and the distribution of MACE among individuals stratified by TyG index quartiles. In the highest TyG index quartile group, the proportion of male patients was lower, while BMI, SBP, and HR were higher. The proportions of patients with DM and AMI, as well as those who underwent PCI, were also higher. Additionally, WBC, NEUT, LYMPH, and PLT counts were elevated, along with higher levels of LDL-C, HDL-C, TG, TC, BUN and FBG. In contrast, the eGFR was lower. Regarding the individual components of MACE, only the rate of rehospitalization for UA was higher in this group. No statistically significant differences were observed among the four groups with respect to the remaining variables, with all *P* values exceeding 0.05.

**Table 2:** Baseline characteristics according to quartiles of the TyG index.

Characteristic	Overall	Q1	Q2	Q3	Q4	*p*-value
	*N* = 1,024	*N* = 256	*N* = 256	*N* = 256	*N* = 256	
Age, years	67 (60, 72)	67 (61, 73)	67 (60, 71)	67 (60, 73)	67 (59, 71)	0.351
Male, *N* (%)	664 (64.8)	199 (77.7)	167 (65.2)	156 (60.9)	142 (55.5)	<0.001
BMI, kg/m^2^	26.00 (24.20, 28.30)	25.30 (23.53, 27.18)	26.00 (24.13, 28.40)	26.00 (24.20, 28.38)	26.85 (24.93, 29.18)	<0.001
SBP, mmHg	137 (125, 148)	136 (124, 145)	137 (125, 149)	138 (125, 149)	139 (127, 150)	0.042
DBP, mmHg	78 (70, 85)	76 (69, 85)	78 (71, 85)	78 (70, 85)	78 (71, 86)	0.092
HR, bpm	68 (61, 76)	66 (59, 73)	68 (61, 75)	69 (60, 76)	71 (63, 78)	<0.001
Smoking, *N* (%)	323 (31.5)	85 (33.2)	77 (30.1)	88 (34.4)	73 (28.5)	0.454
Drinking, *N* (%)	243 (23.7)	68 (26.6)	60 (23.4)	57 (22.3)	58 (22.7)	0.656
Residence, *N* (%)						0.249
Local	608 (59.4)	165 (64.5)	145 (56.6)	152 (59.4)	146 (57.0)	
Non-local	416 (40.6)	91 (35.5)	111 (43.4)	104 (40.6)	110 (43.0)	
DM, *N* (%)	396 (38.7)	59 (23.0)	88 (34.4)	95 (37.1)	154 (60.2)	<0.001
AMI, *N* (%)	269 (26.3)	46 (18.0)	74 (28.9)	71 (27.7)	78 (30.5)	0.005
Number of coronary lesions, *N* (%)						0.842
One-vessel disease	99 (9.7)	29 (11.3)	24 (9.4)	23 (9.0)	23 (9.0)	
Two-vessel disease	196 (19.1)	52 (20.3)	49 (19.1)	52 (20.3)	43 (16.8)	
Multi-vessel disease	729 (71.2)	175 (68.4)	183 (71.5)	181 (70.7)	190 (74.2)	
PCI, *N* (%)	497 (48.5)	102 (39.8)	126 (49.2)	122 (47.7)	147 (57.4)	0.001
Length of hospital stay, days	4 (2, 5)	3 (2, 5)	4 (3, 5)	3 (2, 5)	4 (3, 5)	0.251
LVEF, %	62 (60, 64)	62 (60, 64)	62 (60, 64)	62 (60, 64)	62 (60, 64)	0.815
WBC, 10^9^/L	6.52 (5.47, 7.74)	6.07 (5.14, 7.15)	6.53 (5.35, 7.70)	6.74 (5.62, 7.98)	6.94 (5.81, 8.12)	<0.001
NEUT, 10^9^/L	3.88 (3.07, 4.91)	3.57 (2.78, 4.37)	3.90 (2.98, 5.08)	3.88 (3.10, 5.13)	4.19 (3.36, 5.13)	<0.001
LYMPH, 10^9^/L	1.83 (1.48, 2.27)	1.70 (1.41, 2.11)	1.78 (1.44, 2.22)	1.89 (1.58, 2.33)	1.97 (1.60, 2.38)	<0.001
MONO, 10^9^/L	0.49 (0.40, 0.61)	0.48 (0.40, 0.60)	0.50 (0.41, 0.61)	0.50 (0.40, 0.63)	0.49 (0.41, 0.61)	0.612
PLT, 10^9^/L	217 (184, 258)	203 (173, 240)	214 (181, 257)	220 (189, 267)	227 (193, 263)	<0.001
TC, mmol/L	4.19 (3.46, 5.05)	3.70 (3.13, 4.32)	3.86 (3.23, 4.75)	4.40 (3.71, 5.11)	4.79 (3.96, 5.81)	<0.001
TG, mmol/L	1.24 (0.91, 1.79)	0.77 (0.63, 0.89)	1.11 (0.94, 1.23)	1.52 (1.28, 1.77)	2.29 (1.84, 3.03)	<0.001
HDL-C, mmol/L	1.34 (1.17, 1.54)	1.41 (1.20, 1.61)	1.35 (1.17, 1.55)	1.35 (1.18, 1.55)	1.30 (1.14, 1.50)	0.011
LDL-C, mmol/L	2.26 (1.72, 2.99)	1.95 (1.50, 2.44)	2.10 (1.67, 2.89)	2.47 (1.97, 3.13)	2.6 (2.00, 3.41)	<0.001
FBG, mmol/L	5.56 (4.95, 6.83)	5.00 (4.60, 5.45)	5.46 (4.92, 6.23)	5.71 (5.14, 6.76)	7.13 (5.74, 8.66)	<0.001
BUN, mmol/L	5.95 (4.98, 7.15)	5.73 (4.97, 6.90)	5.86 (4.87, 7.00)	6.04 (5.03, 7.07)	6.19 (5.01, 7.89)	0.023
Scr, umol/L	95 (86, 107)	95 (86, 106)	94 (85, 106)	97 (87, 109)	95 (85, 107)	0.345
eGFR, mL/min/1.73 m^2^	65.90 (57.76, 74.25)	67.66 (61.12, 75.72)	66.05(58.66, 77.57)	64.06(56.54, 71.42)	65.21(56.28, 73.61)	0.001
NT-proBNP, pg/mL	108 (45, 416)	104 (48, 353)	120 (47, 515)	106 (44, 440)	99 (41, 396)	0.545

BMI, body mass index; SBP, systolic blood pressure; DBP, diastolic blood pressure; HR, heart rate; DM, diabetes mellitus; AMI, acute myocardial infarction; PCI, percutaneous coronary intervention; LVEF, left ventricular ejection fraction; WBC, white blood cell; NEUT, neutrophil; LYMPH, lymphocyte; MONO, monocyte; PLT, platelet; TC, total cholesterol; TG, triglycerides; HDL-C, high-density lipoprotein cholesterol; LDL-C, low-density lipoprotein cholesterol; FBG, fasting blood glucose; BUN, blood urea nitrogen; Scr, Serum Creatinine; eGFR, estimated glomerular filtration rate; NT-proBNP, N-terminal pro-B-type natriuretic peptide; TyG index, Triglyceride-glucose index.

### 3.3 Association between the TyG index and 2-year MACE

**Figure 2:**
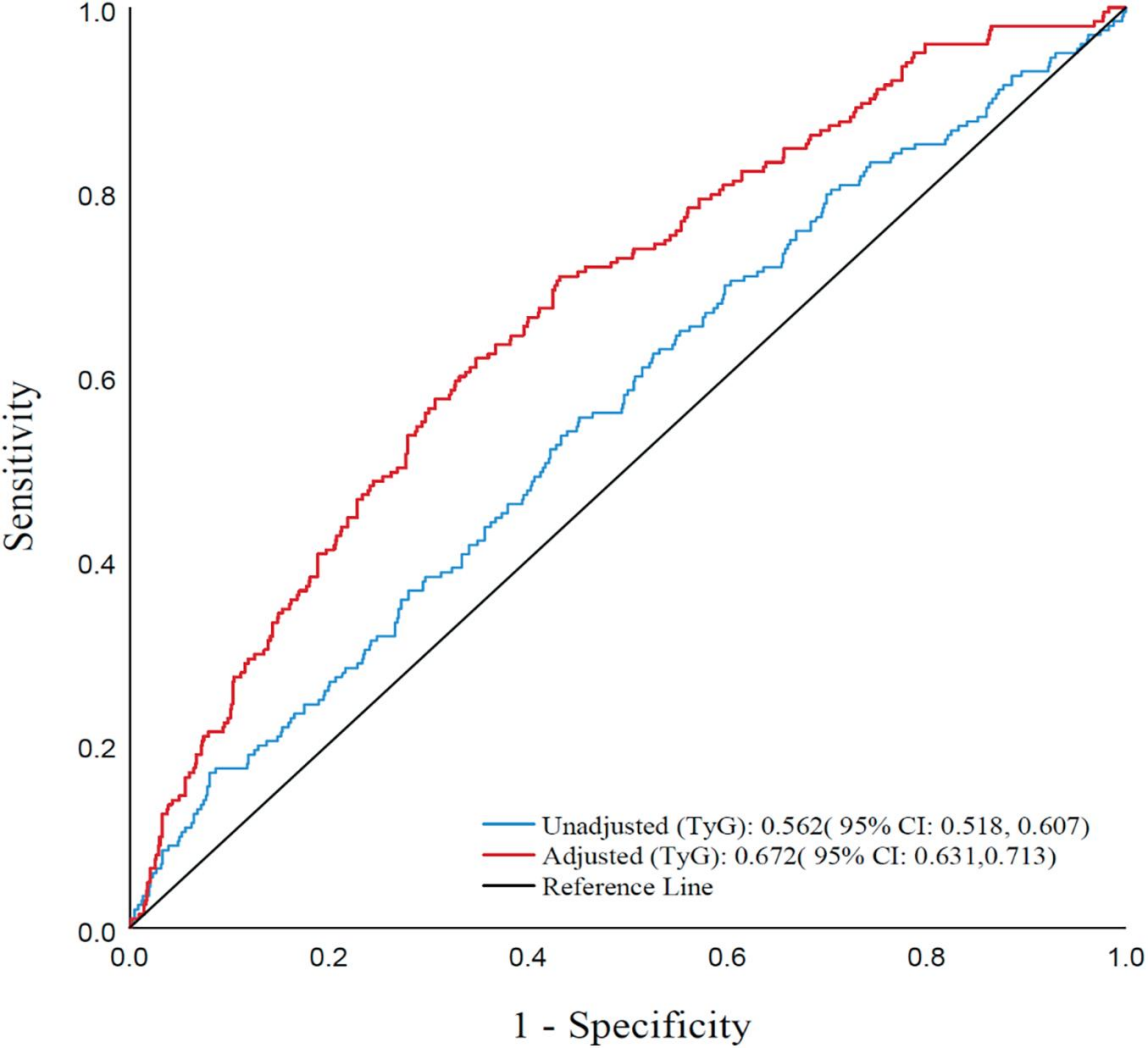
Schematic diagram of the ROC curve and AUC values.

**Figure 3:**
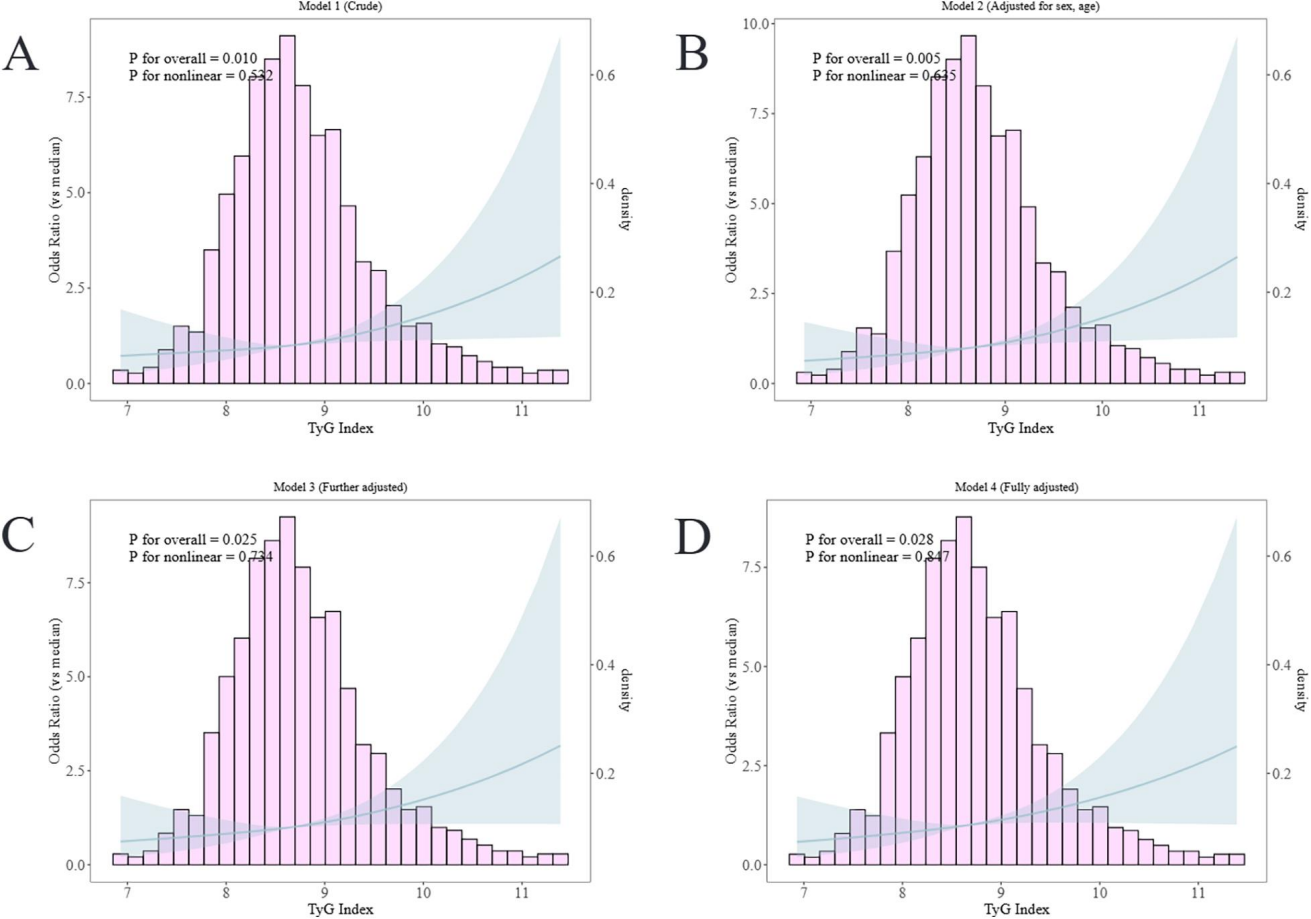
Restricted cubic spline models.

Table 3 presents the association between the TyG index and the risk of MACE. Multivariable logistic regression models showed a significant positive association between the TyG index and 2-year MACE in individuals with OCAD and hypertension. After full adjustment for all covariates in Model 4, each one-unit increase in the TyG index was associated with a 45.2% increased risk of MACE (OR: 1.452, 95% CI 1.101–1.913; *P* = 0.008). Further regression analysis categorizing the TyG index into quartiles revealed that in Models 1–3, the highest quartile (Q4) demonstrated a significant association with MACE risk (*P* < 0.05). In Model 4, however, both the Q3 and Q4 quartiles showed a significant association with MACE risk compared with the lowest quartile (Q1) (OR for Q3: 1.707, 95%CI 1.028–2.835, *P* = 0.039; OR for Q4: 1.953, 95%CI 1.140–3.345, *P* = 0.015). The TyG index was strongly linked with rehospitalization for UA, both as a continuous variable (OR: 2.047, 95% CI 1.435–2.921; *P* < 0.001) and when categorized. However, no statistically significant association was observed with the other secondary endpoints (Supplementary Tables S3–S6). Supplementary Table S7 presents the correlation between TyG-BMI and MACE. After full adjustment for confounders, the OR was 1.014 (95% CI: 1.004, 1.025, *P* = 0.007). However, when TyG-BMI was entered into the model as a categorical variable, the association was not statistically significant. In addition, ROC curve indicated that the fully adjusted model that contained the TyG index had better predictive performance than the unadjusted model, with an AUC of 0.672 (0.631, 0.713) vs. 0.562 (0.518, 0.607) (Figure 2). The AUC for TyG-BMI was similar to that of the TyG index (Supplementary Figure S2). RCS curve revealed a significant positive linear relationship between the TyG index and risk of MACE in patients with hypertension and OCAD (Figure 3).

**Table 3:** Logistic regression models analyzing the relationship between TyG index and MACE.

Characteristic	Model 1	Model 2	Model 3	Model 4
	OR (95%CI) *P*	OR (95%CI) *P*	OR (95%CI) *P*	OR (95%CI) *P*
TyG index	1.419 (1.122, 1.796) 0.004	1.481 (1.165, 1.884) 0.001	1.385 (1.065, 1.802) 0.015	1.452 (1.101, 1.913) 0.008
TyG				
Quartile 1	1 (Reference)	1 (Reference)	1 (Reference)	1 (Reference)
Quartile 2	1.509 (0.950, 2.396) 0.081	1.578 (0.990, 2.515) 0.055	1.482 (0.916, 2.396) 0.109	1.581 (0.968, 2.582) 0.068
Quartile 3	1.509 (0.950, 2.396) 0.081	1.598 (1.000, 2.551) 0.050	1.506 (0.927, 2.446) 0.098	1.707 (1.028, 2.835) 0.039
Quartile 4	1.852 (1.179, 2.909) 0.008	2.006 (1.263, 3.186) 0.003	1.715 (1.038, 2.832) 0.035	1.953 (1.140, 3.345) 0.015

Model 1: Unadjusted.

Model 2: Adjusted sex, age.

Model 3: Further adjusted for residence, diabetes mellitus (DM), acute myocardial infarction (AMI), number of coronary lesions, percutaneous coronary intervention (PCI), smoking history, alcohol consumption history, length of hospital stay, systolic blood pressure (SBP), diastolic blood pressure (DBP), heart rate (HR), body mass index (BMI).

Model 4: Additionally adjusted for left ventricular ejection fraction (LVEF), platelet (PLT), high-density lipoprotein cholesterol (HDL-C), low-density lipoprotein cholesterol (LDL-C), blood urea nitrogen (BUN), estimated glomerular filtration rate (eGFR), N-terminal pro-B-type natriuretic peptide (NT-proBNP).

### 3.4 Subgroup analysis

**Figure 4:**
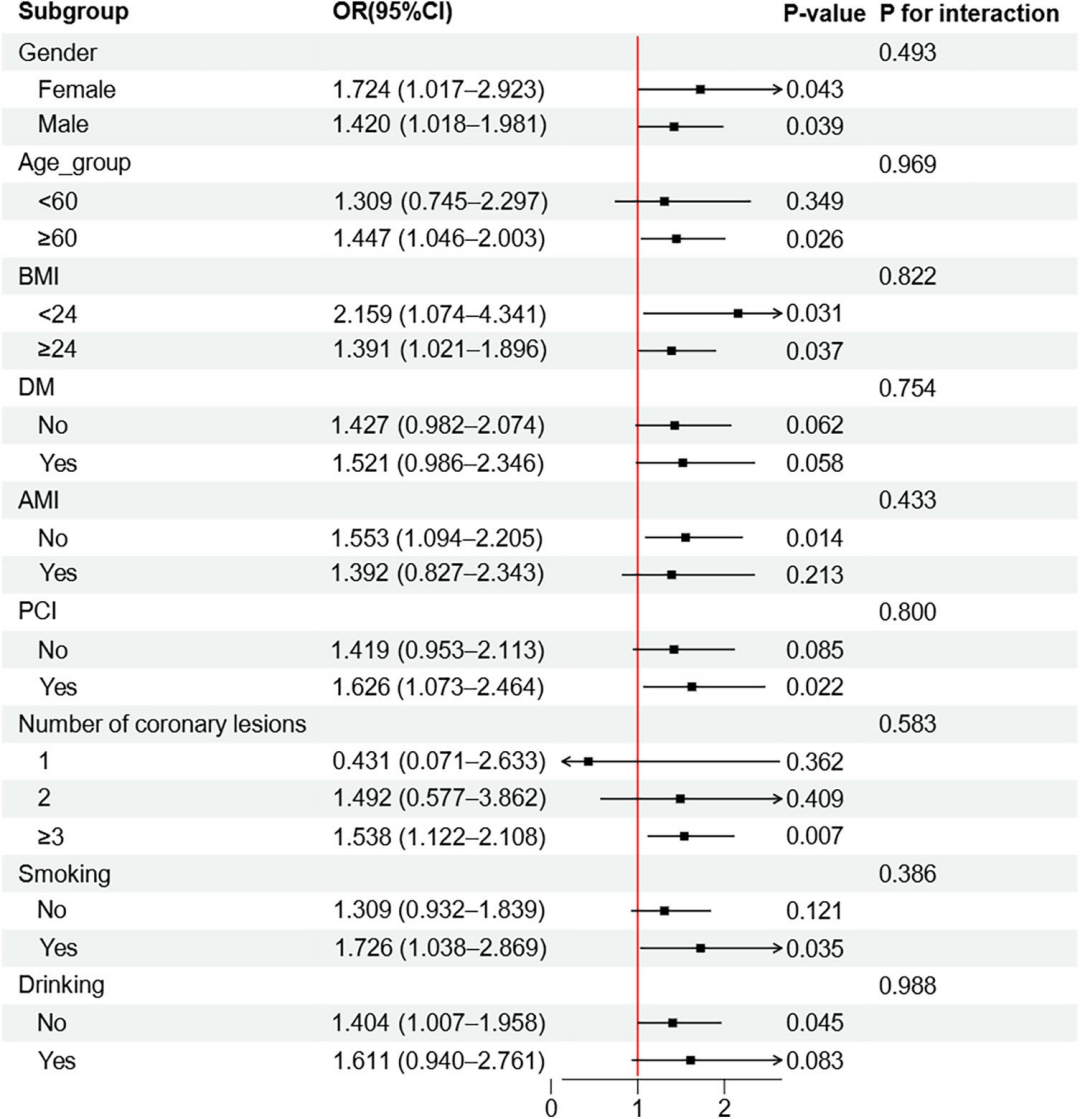
TyG index and MACE subgroup analysis.

Subgroup analyses were conducted according to sex, age, BMI, presence of DM, diagnosis of AMI, performance of PCI, number of coronary lesions (1, 2, or multivessel), smoking history, and alcohol consumption history, with adjustment for covariates. Figure 4 presents the results of subgroup analyses regarding the association between the TyG index and 2-year MACE in patients with hypertension and OCAD. The results indicated that the association between the TyG index and MACE risk was not significantly modified by these variables. Analyses of the secondary endpoints yielded similar findings (Supplementary Tables S8–S11). The subgroup analysis results for TyG-BMI were consistent with those for the TyG index, and the findings were consistent across all subgroups (Supplementary Table S12).

### 3.5 MR analysis

**Figure 5:**
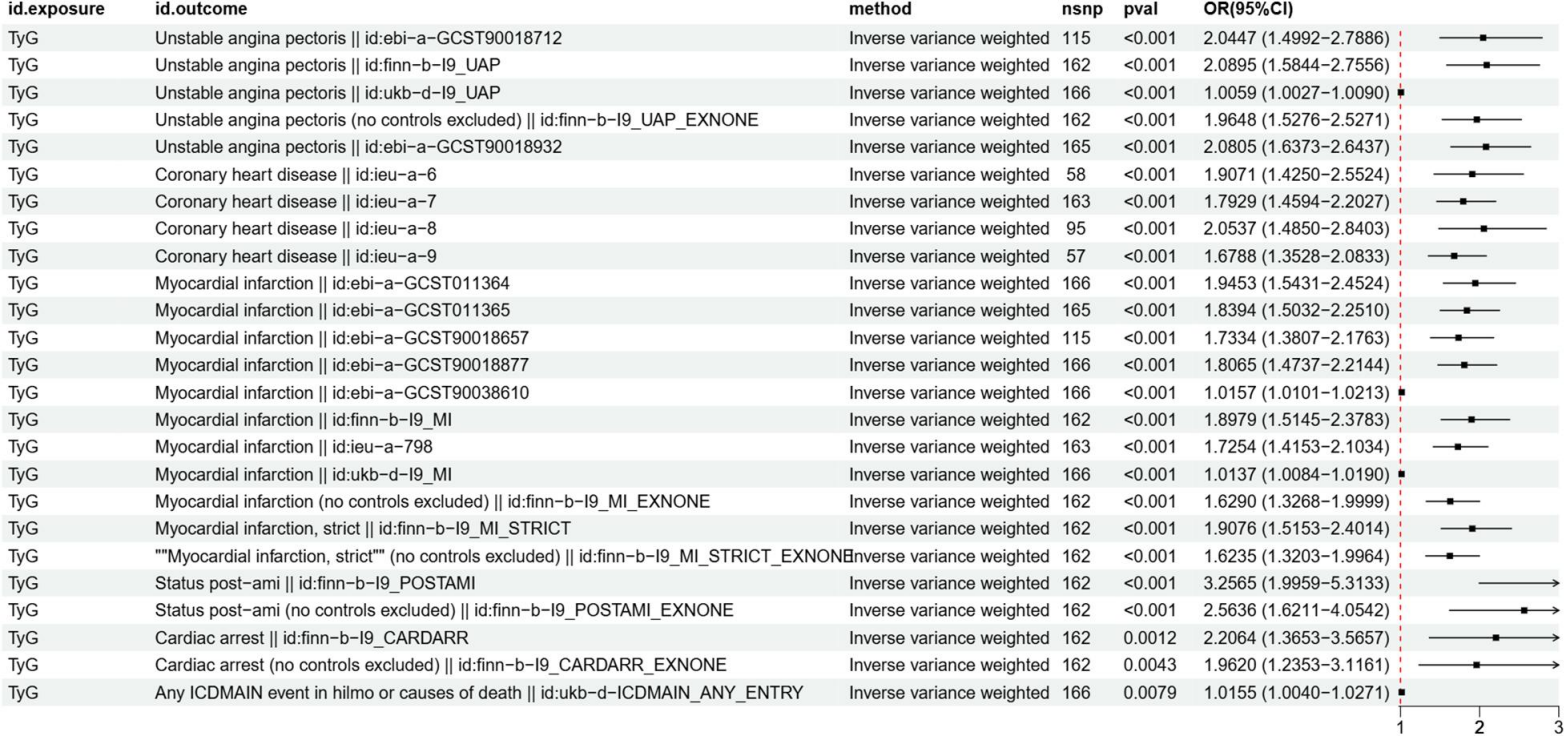
Forest plot of MR analysis.

Figure 5 presents evidence of a significant association between the TyG index and the occurrence of UA, coronary heart disease, MI, cardiac arrest, and all-cause death. The IVW *P* values for UA (ebi−a−GCST90018712, finn−b−I9_UAP, ukb−d−I9_UAP, finn−b−I9_UAP_EXNONE and ebi−a−GCST90018932), coronary heart disease (ieu−a−6, ieu−a−7, ieu−a−8 and ieu−a−9) and MI (ebi−a−GCST011364, ebi−a−GCST011365, ebi−a−GCST90018657, ebi−a−GCST90018877, ebi−a−GCST90038610, finn−b−I9_MI, ieu−a−798, ukb−d−I9_MI, finn−b−I9_MI_EXNONE, finn−b−I9_MI_STRICT, finn−b−I9_MI_STRICT_EXNONE, finn−b−I9_POSTAMI and finn−b−I9_POSTAMI_EXNONE) were all less than 0.001. the corresponding IVW-derived *P*-values for cardiac arrest (finn−b−I9_CARDARR and finn−b−I9_CARDARR_EXNONE) and all-cause death (ukb−d−ICDMAIN_ANY_ENTRY) were all below 0.05.

## 4 Discussion

In this retrospective cohort of patients with hypertension and OCAD, a higher TyG index was independently associated with increased 2-year MACE risk: each one-unit increase conferred 45.2% higher odds (OR: 1.452; *P* = 0.008), and the highest quartile had nearly double the risk vs. the lowest (OR: 1.953; *P* = 0.015). The association was linear and consistent across subgroups by sex, age, diabetes, PCI status, and coronary disease severity. MR further supported a potential causal role of elevated TyG index in MI, UA, coronary heart disease, cardiac arrest, and all-cause mortality. These results highlight the importance of incorporating the TyG index into cardiovascular risk assessment and its promise as a biomarker and therapeutic target in high-risk multimorbid populations.

Mounting evidence demonstrated that the TyG index is a predictor of adverse prognosis in CVD, including hypertension and CAD (16). A large-scale meta-analysis demonstrated that people with an elevated TyG index have a higher CAD risk, more severe coronary artery lesions, and a worse prognosis (30). Tao et al. showed that the TyG index is a potential predictor of 1-year MACE in individuals with hypertension and CAD (31). Similarly, Liu et al. reported consistent findings (32). Moreover, Pan et al. demonstrated that the TyG index has a J-shaped relationship with the incidence of OCAD in hypertensive patients (33). However, the relationship between the TyG index and long-term adverse prognosis in patients with hypertension and OCAD remains unclear. Even after extensive adjustment for confounders, the TyG index remained a significant predictor of adverse outcomes in this multimorbid population. Although we conducted a longer follow-up period, our findings remained consistent with prior research. Furthermore, by employing MR analysis, we effectively addressed reverse causation and minimized the impact of unmeasured confounders, thereby offering strong corroboration of our findings. Of note, some previous studies observed significant interaction effects across different subgroups (34, 35); however, this finding was not replicated in our study. For example, Wang et al. found that there were significant interaction effects in the relationship between the TyG index and MACE across different subgroups, including age, sex, DM status, and whether PCI was performed (34). Subgroup analysis of this study showed that the relationship between the TyG index and the 2-year risk of MACE in patients with hypertension and OCAD was not significantly modified by sex, age, BMI, presence of DM, diagnosis of AMI, performance of PCI, number of coronary lesions, smoking history, or alcohol consumption history. The TyG index was independently associated with MACE in both sexes; however, no significant interaction by sex was detected. Furthermore, the TyG index showed no independent association with the MACE risk, irrespective of DM status (all *P* > 0.05). This finding was also supported by interaction testing, which showed no significant interaction between patients with and without DM. Thus, we found that predictive capacity of the TyG index was unaffected by sex, age, and DM status, which aligns with findings from several previous studies (36–38). These findings indicate that the TyG index consistently predicts long-term adverse outcomes across subgroups, supporting its robustness for risk stratification. When categorized, Q4 patients had significantly higher risk than Q1, warranting more aggressive lifestyle and pharmacologic interventions. Although TyG-BMI showed a lower odds ratio than the TyG index, both had similar AUCs. This implies that the TyG index could be a more meaningful predictor of MACE than TyG-BMI. Cui et al. found that the TyG index was superior to its modified indices in identifying the risk of cardiovascular events, a finding that is in agreement with our study results (39). Therefore, for high-risk patients with this comorbidity, paying more attention to the TyG index is more important than to its modified indices. RCS analysis based on logistic regression models exhibited a significant positive linear correlation between the TyG index and MACE. Furthermore, MR results indicated that increased TyG index levels were associated with a significantly higher risk of various adverse cardiovascular outcomes including UA, coronary heart disease, MI, all-cause death, and cardiac arrest. This provides genetic evidence support for our study.

Previous studies have demonstrated that incorporating the TyG index into a prediction model based on the Global Registry of Acute Coronary Events (GRACE) score can significantly improve the predictive value for the risk of MACE in patients with ST-segment elevation myocardial infarction (STEMI) (40, 41). Particularly in patients with STEMI and chronic kidney disease (CKD), the TyG index demonstrates a more pronounced improvement in the predictive ability of the GRACE score compared with other IR indicators (40). Huang et al. found that combining the TyG index with B-type natriuretic peptide (BNP) can more effectively predict the risk of MACE in patients with STEMI (42). These observations underscore the clinical utility of routine TyG index assessment to facilitate timely detection and management among high-risk populations, which may contribute to reducing the mortality and incidence of MACE in individuals with hypertension and OCAD. Furthermore, they offer new insights into how the TyG index influences adverse prognosis in this patient population. IR, as represented by the TyG index, is a key mechanism explaining its association with adverse cardiovascular prognosis (43). Previous studies have shown that the TyG index, as a reliable surrogate marker of IR, has predictive capabilities comparable to, or even better than, traditional surrogate markers, including homeostasis model assessment of IR (HOMA-IR) (44–46). A study of American adults confirmed that the TyG index is more effective than HOMA-IR in assessing IR (45). In addition, the TyG index also exhibited superior performance in predicting MACE. For example, when assessing the prognosis of patients with cardiometabolic multimorbidity, the TyG index is significantly superior to HOMA-IR (46, 47). IR is implicated in various mechanisms and pathways in CVD, including inflammatory responses, oxidative stress, disorders of glucose and lipid metabolism, endothelial injury and dysfunction, and damage to the mitochondrial oxidative respiratory chain (48, 49).

The biological plausibility of the TyG index as a predictor of cardiovascular risk is further supported by the well-established pathophysiological roles of IR in cardiovascular disease. First, under conditions of IR, the body exhibits lipid overflow and ectopic deposition. This lipotoxic effect promotes endothelial dysfunction and the formation of atherosclerotic plaques (50). Concurrently, reduced energy uptake of glucose by cardiomyocytes leads to increased oxidation of free fatty acids and a decrease in cardiac efficiency (51). Second, IR exacerbates cytotoxic effects by inducing the inactivation of nitric oxide (NO), thereby promoting vasoconstriction and endothelial injury (46). Third, IR can directly activate the mitochondrial oxidative respiratory chain, inducing excessive production of oxidative stress and indirectly contributing to endothelial injury and dysfunction (52). Endothelial dysfunction is one of the core pathological mechanisms underlying both hypertension and atherosclerosis (53). Furthermore, IR induces platelet hyperactivation and an abnormal increase in thromboxane A2 (TXA2), consequently leading to thrombus formation (54). Finally, IR, often accompanied by hyperglycemia, hyperlipidemia, and hyperinsulinemia, indirectly leads to endoplasmic reticulum stress and excessive production of reactive oxygen species (ROS). This disrupts calcium homeostasis, inducing apoptosis, necrosis, and autophagy of cardiomyocytes, and ultimately results in systolic and diastolic dysfunction (55). The aforementioned mechanisms have collectively contributed to the onset and progression of atherosclerosis, promoting plaque formation and reducing plaque stability, thereby significantly increasing the risk of MACE. Notably, the TyG index, composed of FBG and TG, is distinguished by its efficiency and simplicity, making it suitable for widespread clinical application, particularly in large-scale population screening. Monitoring the TyG index may enable the early detection of individuals at high risk and facilitate the implementation of necessary early prevention and treatment strategies, thereby alleviating the health and economic burden on society as a whole. This study provides valuable insights for risk management in patients with hypertension and OCAD.

This study has several key strengths. First of all, we demonstrated for the first time the relationship of the TyG index with long-term outcomes in people with coexisting hypertension and OCAD. Second, we found that the correlation between the TyG index and MACE risk was consistent across different subgroups, demonstrating its broad generalizability and robustness as a risk prediction tool. Lastly, we applied MR analysis to validate a potential cause-effect relationship between them, thereby addressing the limitations of observational studies arising from confounding factors and reverse causation.

Several limitations of this study should be considered. To begin with, this was a single-center, retrospective observational study, which may have introduced potential selection bias; therefore, caution is needed when extrapolating these findings to other regions or ethnic groups. Second, we did not incorporate lifestyle factors such as medication use, nutrition, and physical activity, and therefore could not adjust for these confounding factors. Subsequently, the absence of insulin data precluded a direct comparison of the TyG index with HOMA-IR. Although the variables collected in our study partially overlapped with those included in the GRACE score, the GRACE score was specifically designed for patients with ACS. Since this study consecutively enrolled hospitalized patients with CAD without distinguishing between ACS and chronic coronary syndrome (CCS), the incremental predictive value of the TyG index over the GRACE score could not be validated in this population. Moreover, laboratory measurements were only collected at baseline, which precludes an assessment of the effect of cumulative exposure to the TyG index on the outcomes. Finally, while several biological mechanisms linking the TyG index to 2-year MACE in patients with hypertension and OCAD have been proposed, the underlying pathways remain incompletely understood. Further basic and multicenter prospective studies that track the longitudinal trajectory of the TyG index are needed to clarify these mechanisms and strengthen the evidence base.

## 5 Conclusion

This study found that elevated TyG index levels were strongly associated with worse clinical outcomes in individuals with hypertension and OCAD, and this association remained consistent across different subgroups. The potential causal relationship between them was further supported by MR analysis. Therefore, in clinical practice, close attention should be paid to the TyG index in such patients, and early intervention and management should be intensified for this high-risk population with comorbidity to improve their prognosis.

## Data Availability

The datasets analysed during the current study are not publicly available due to protecting participants' privacy, but are available from the corresponding author upon reasonable request. Requests to access these datasets should be directed to Haichu Yu, haichuyu@163.com
