# Supplementary figures and images for: TyG index predicts adverse cardiovascular outcomes in patients with multimorbidity of hypertension and obstructive coronary artery disease: a cohort study
Source: Front Cardiovasc Med. 2026 Jul 16;13:1861084. doi: 10.3389/fcvm.2026.1861084 (PMC13422514; doi:10.3389/fcvm.2026.1861084)

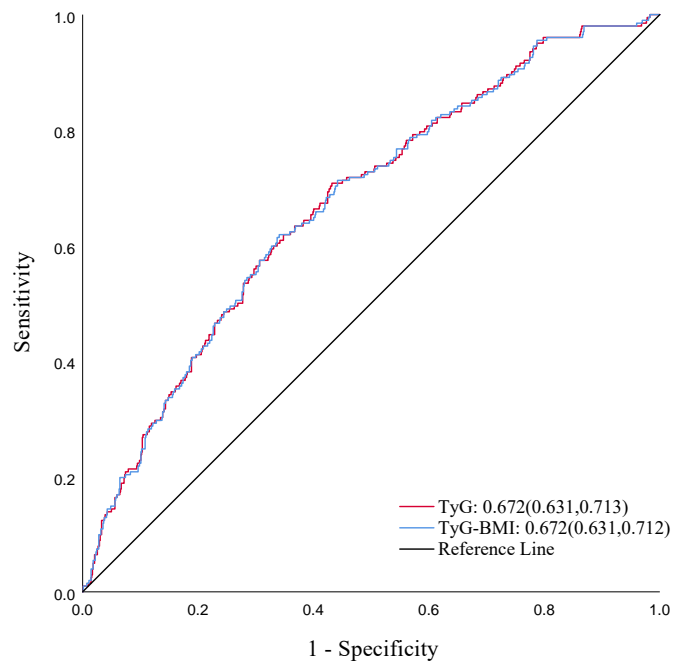

Supplement: Supplementary file 2 [file Image2.pdf]
