# Supplementary material for: TyG index predicts adverse cardiovascular outcomes in patients with multimorbidity of hypertension and obstructive coronary artery disease: a cohort study
Source: Front Cardiovasc Med. 2026 Jul 16;13:1861084. doi: 10.3389/fcvm.2026.1861084 (PMC13422514; doi:10.3389/fcvm.2026.1861084)
Supplement: Supplementary file 4 [file Table2.docx]

**Supplementary Table S2** Detailed distribution of MACE across TyG index quartile groups

| **Characteristic** | **Overall N=1,024** | **Q1 N=256** | **Q2 N=256** | **Q3 N=256** | **Q4 N=256** | ***p*-value** |
| --- | --- | --- | --- | --- | --- | --- |
| MACE, N (%) | 202(19.73) | 37(14.45) | 52(20.31) | 52(20.31) | 61(23.83) | 0.062 |
| all-cause death, N (%) | 14(1.37) | 5(1.95) | 3(1.17) | 5(1.95) | 1(0.39) | 0.364 |
| non-fatal MI, N (%) | 32(3.13) | 10(3.91) | 7(2.73) | 7(2.73) | 8(3.13) | 0.856 |
| unplanned revascularization, N (%) | 57(5.57) | 9(3.52) | 19(7.42) | 13(5.08) | 16(6.25) | 0.255 |
| rehospitalization for UA, N (%) | 99(9.67) | 13(5.08) | 23(8.98) | 27(10.55) | 36(14.06) | 0.007 |

Abbreviations: TyG, triglyceride-glucose index; MACE, major adverse cardiovascular events; MI, myocardial infarction; UA: unstable angina.
