# Supplementary material for: TyG index predicts adverse cardiovascular outcomes in patients with multimorbidity of hypertension and obstructive coronary artery disease: a cohort study
Source: Front Cardiovasc Med. 2026 Jul 16;13:1861084. doi: 10.3389/fcvm.2026.1861084 (PMC13422514; doi:10.3389/fcvm.2026.1861084)
Supplement: Supplementary file 9 [file Table7.docx]

**Supplementary Table S7** Logistic regression models analyzing the relationship between TyG-BMI and MACE

| **Characteristic** | **Model 1**  **OR (95%CI) P** | **Model 2**  **OR (95%CI) P** | **Model 3**  **OR (95%CI) P** | **Model 4**  **OR (95%CI) P** |
| --- | --- | --- | --- | --- |
| TyG-BMI index | 1.006 (1.002,1.011) 0.004 | 1.007 (1.003,1.012) 0.002 | 1.103 (1.003,1.023) 0.013 | 1.014 (1.004,1.025) 0.007 |
| TyG-BMI |  |  |  |  |
| Quartile 1 | 1 (Reference) | 1 (Reference) | 1 (Reference) | 1 (Reference) |
| Quartile 2 | 1.116 (0.705,1.767) 0.639 | 1.132 (0.714,1.794) 0.598 | 1.138 (0.682,1.900) 0.621 | 1.198 (0.711,2.019) 0.497 |
| Quartile 3 | 1.206 (0.766,1.900) 0.419 | 1.244 (0.788,1.965) 0.348 | 1.297 (0.716,2.347) 0.391 | 1.336 (0.726,2.459) 0.352 |
| Quartile 4 | 1.734 (1.123,2.677) 0.013 | 1.867 (1.189,2.931) 0.007 | 1.976 (0.930,4.201) 0.077 | 2.165 (0.994,4.713) 0.052 |

Model 1: Unadjusted

Model 2: Adjusted sex, age

Model 3: Further adjusted for residence, diabetes mellitus (DM), acute myocardial infarction (AMI), number of coronary lesions, percutaneous coronary intervention (PCI), smoking history, alcohol consumption history, length of hospital stay, systolic blood pressure (SBP), diastolic blood pressure (DBP), heart rate (HR), body mass index (BMI)

Model 4: Additionally adjusted for left ventricular ejection fraction (LVEF), platelet (PLT), high-density lipoprotein cholesterol (HDL-C), low-density lipoprotein cholesterol (LDL-C), blood urea nitrogen (BUN), estimated glomerular filtration rate (eGFR), N-terminal pro-B-type natriuretic peptide (NT-proBNP)
